# Does luteal phase support in MOH-IUI treatment improve cumulative live birth rates in couples with unexplained subfertility? Study protocol of the LUMO study: a multicentre, randomised, double-blind, controlled trial with cost-effectiveness analysis

**DOI:** 10.1136/bmjopen-2025-111872

**Published:** 2025-11-19

**Authors:** Emma Preesman, Katja Drechsel, Heleen Crommelin, Frank Broekmans, Willem Verpoest, Simone Broer, On behalf of the LUMO Study Group, Anna Musters

**Affiliations:** 1 Division Woman and Baby, University Medical Centre Utrecht, Utrecht, The Netherlands; 2 Laboratories, Pharmacy, and Biomedical Genetics, University Medical Centre Utrecht, Utrecht, The Netherlands

**Keywords:** Reproductive medicine, Subfertility, Randomized Controlled Trial

## Abstract

**Introduction:**

Couples diagnosed with unexplained subfertility are advised to start mild ovarian hyperstimulation and intrauterine insemination (MOH-IUI) as a primary treatment. Natural feedback mechanisms and hormone release are affected by artificially stimulated cycles and induced ovulation. Additional luteal support could positively affect progesterone patterns in the luteal phase. The LUMO study evaluates whether the addition of exogenous progesterone in the luteal phase following MOH-IUI treatment cycle will improve pregnancy and live birth rates.

**Methods and analysis:**

A multicentre randomised, double-blind, controlled trial will be conducted in Dutch fertility clinics, academic and non-academic hospitals. There are two treatment arms: group A progesterone luteal phase support; group B placebo, without crossover. All initiated MOH-IUI cycles within 6 months after randomisation are included (study period). Participants will start study medication, applying a daily dosage of 2dd 300 mg progesterone (Utrogestan) or 2dd 300 mg placebo in vaginal capsules on the second day after the IUI procedure. Treatment is continued until the onset of menstruation, a negative pregnancy test (IUI+14 days), a miscarriage or until 7 weeks of gestation in case of a viable pregnancy. Follow-up ends at 12 months after the end of study period (18 months after study randomisation). The primary outcome is cumulative pregnancy rate, achieved within 6 months after randomisation, leading to live birth. A total of 1008 patients (504 patients in each group) will be included.

**Ethics and dissemination:**

The study was approved by the Central Committee on Research Involving Human Subjects on 30 January 2023. All participating sites have the approval of the local Board of Directors to participate in the LUMO study. An informed consent form will be signed by all participants. Study results will be presented at (inter)national conferences and published in peer-reviewed journals. It is expected that the results of this trial will be used to draft national guidelines on this issue.

**Trial registration number:**

The study is registered in the EU CTIS trial register (2022-501534-33-00), the Dutch trial registry (registration number: LTR 24508), ClinicalTrials.gov (NCT05080569) and the WHO registry (universal trial number: U1111-1280-9461).

**STRENGTHS AND LIMITATIONS OF THIS STUDY**

- This is the largest randomised trial investigating the efficacy of the addition of luteal phase support in mild ovarian hyperstimulation and intrauterine insemination (MOH-IUI) treatment.
- This trial follows a double-blind, placebo-controlled design to ensure that neither the participant will be influenced in her behaviour by knowing which ‘medication’ she uses, nor the doctor or nurse administering the MOH-IUI treatment is biased in their actions.
- Although the use of micronised progesterone is proven to be safe during (early) pregnancy and the LUMO trial is considered a low-risk clinical trial, participants will receive an online medication diary at the start of every cycle and a questionnaire at the end of the study period about any side effects and other issues to closely track the safety of the study participants.
- Complete adherence to the study protocol cannot be assured as participants are not required to return unused study medication to their study team.

## Introduction

 During the natural female cycle, hormone production is regulated by feedback mechanisms. Follicle-stimulating hormone (FSH) triggers growth and maturation of a dominant follicle during the follicular phase. Oestrogen levels progressively increase and during a final rapid increase, feedback mechanisms occur that elicit a surge of luteinising hormone (LH) and FSH secretion from the pituitary, inducing final maturation and ovulation of the oocyte. Driven by intensified LH release, the corpus luteum produces progesterone during the luteal phase which allows endometrial secretory transformation and promotes endometrial receptivity, critical for implantation of the developing blastocyst and maintenance of (early) pregnancy.^1^,^8^

The hormone release from the developing follicle(s) and the natural feedback mechanisms may become altered in artificially stimulated cycles and induced ovulation. Supraphysiologic serum steroid levels during the ovarian stimulation phase may cause pituitary inhibition, thereby suppressing the natural release of LH.^9^,^11^ The single-dosage human chorionic gonadotropin (hCG) that is injected as an ovulation trigger will induce a stronger pathway than produced by the normal spontaneous LH surge. The corpus luteum is adequately supported by the exogenous LH-like signal during the first days of the luteal phase until this signal is cleared with the resorption of the hCG trigger after approximately 5–6 days. Without additional support, progesterone levels tend to drop considerably and earlier compared with the natural progesterone pattern.^10^ Both the early peak and later defective progesterone secretion may lead to premature or even insufficient arrival into the receptive state of the endometrium and insufficient maintenance of endometrium function thereafter. All this may result in failure of embryo implantation and growth.^12^ Luteal phase support (LPS), by administering exogenous progesterone, has been previously proven to positively affect the level of progesterone and the length of the luteal phase.^13^

Intrauterine insemination (IUI) is a widely used fertility treatment in couples diagnosed with unexplained or mild male subfertility. Mild ovarian hyperstimulation (MOH), applying a low dosage of gonadotropins (eg, FSH), letrozole or clomiphene citrate (CC), is generally used to achieve growth of one or two dominant follicles. A single dose of hCG (often Ovitrelle 250 µg) is used as an exogenous trigger to complete final oocyte maturation and induce ovulation. Subsequently, prewashed semen is injected into the uterus at approximately 24–42 hours after the hCG trigger.^14^ Annually, approximately 9500 couples enter a MOH-IUI infertility treatment programme in the Netherlands (based on Dutch Care Authority (NZA) documentation).^15^

Currently, there is no consensus on the use of progesterone supplementation after MOH-IUI. A meta-analysis by Green *et al* in 2017 concluded that LPS could be effective in MOH-IUI, specifically in mild stimulation cycles using low-dose FSH, with live birth occurring more frequently in patients receiving exogenous progesterone (random-effects modelling, RR 1.76, 95% CI 1.29 to 2.40, p<0.001, number needed to treat 11).^16^ An updated and extended literature search and meta-analysis by Casarramona *et al*, in which four additional studies were included compared with the previous review in 2017, found comparable results to the Green review (single cycle; live birth rate RR 2.47, 95% CI 1.37 to 4.44; three randomised controlled trials (RCTs), 678 participants; cumulative live birth rate for up to six cycles: RR 1.49, 95% CI 1.17 to 1.90; five RCTs, 1027 participants).^17^ For both reviews, the level of evidence had been graded low to moderate. The studies in the meta-analysis are often single-centre based, fail to obtain individual power, were mostly not (double-) blinded, included only a single treatment cycle and/or evaluated various types of luteal support. Due to the overall moderate study quality and small to moderate size of the individual studies, the certainty of the evidence should be considered with caution and broad implementation of progesterone luteal support in MOH-IUI treatment cannot be justified with the current research data. The substandard evidence quality warrants a large, well-designed, well-powered trial to provide the evidence that is needed to confirm or dismiss the effect of progesterone LPS after MOH-IUI. For this purpose, the LUMO (LUteal phase support in Mild Ovarian hyperstimulation (MOH) for intra-unterine insemination (IUI)) study was designed, funded and is currently ongoing.

The primary aim of the LUMO study is to determine whether the addition of exogenous progesterone in the luteal phase of MOH-IUI treatment will lead to increased live birth rates and will provide the basis for application of this strategy in daily practice. The hypothesis is that the chance of pregnancy leading to live birth within a 6-month study period will increase from 30% to 39% after MOH-IUI treatment with LPS. In addition, the study seeks to evaluate the total budget impact of the addition of LPS in MOH-IUI treatment in terms of reduction in total budget spent on (in)fertility care through a cost-effectiveness analysis.

## Methods and analysis

### Design and setting

The LUMO study is a multicentre, double-blind, randomised-controlled trial with cost-effectiveness analysis. It is coordinated by the UMC Utrecht, Netherlands, and will be performed within the Dutch Consortium for Healthcare Evaluation and Research in Obstetrics and Gynecology. There are two treatment arms (A LPS; B placebo, non-crossover). All initiated MOH-IUI cycles within 6 months after randomisation are included (study period). Follow-up period ends 12 months after the end of the study period (18 months after randomisation). The primary outcome is cumulative pregnancy, achieved within 6 months after randomisation, leading to live birth rate. The aim is to include 1008 patients (504 patients in each group).

At present, there are 33 sites (fertility clinics, academic and non-academic hospitals) participating in the LUMO study. All participating sites and their corresponding local principal investigator are mentioned in the ‘Acknowledgements’ section.

### Eligibility criteria

Eligibility criteria are in line with the indication for MOH-IUI treatment and in accordance with current (Dutch) NVOG (Nederlandse Vereniging voor Obstetrie en Gynaecologie (Dutch Sciety for Obestrics and Gynaecology)guidelines.^18^ In the Netherlands, in couples with unexplained subfertility, the prognosis for spontaneous pregnancy in the year following the subfertility workup is calculated using the validated Hunault prognostic model.^19^ This model uses the predictive factors female age, subfertility duration, type of subfertility (primary vs secondary), sperm motility and referral status. When the likelihood of spontaneous pregnancy is <30% (or ≥30% after an expectant management period of at least six additional months), MOH-IUI is suggested as the primary treatment. To participate in the study, a participant must meet all of the following criteria: (1) diagnosis of unexplained subfertility; (2) Hunault <30% (or ≥30%, after an expectant management period of at least six additional months); (3) female age 18–43 years; (4) female body mass index (BMI) <45 kg/m^2^; (5) primary or secondary subfertility, for at least a period of 1 year (or, in case of secondary infertility with previous successful MOH-IUI treatment, an expectant management of at least 6 months); (6) regular menstrual cycle; (7) total motile sperm count >10 million (prewash/unprocessed) and (8) first MOH-IUI cycle (with gonadotropins), with the intent to receive this treatment for at least 6 months. Exclusion criteria are (1) uncorrected uterine factors, such as endometrial polyps or submucosal fibroids; (2) insufficient knowledge or understanding of the Dutch or English language and not willing or able to receive study information via a certified translator; (3) not able or willing to provide (written) informed consent and (4) contraindications for vaginal progesterone, particularly females with allergy to peanuts or soya.

### Study procedures

#### Recruitment and randomisation

Potential participants can be informed about the existence of this trial by their treating doctor if they meet eligibility criteria. For additional information, patients will be referred to the websites of UMC Utrecht, Freya (patient federation) and Zorgevaluatie Nederland. More extensive information will be included in the patient information form (PIF) that patients will receive as they expressed their potential interest in participating. After receiving the PIF, the research nurse or researcher of the participating research site will contact potential participants to check for any remaining questions and ask if they indeed want to participate. After obtaining written informed consent (signed by both the female patient and her partner), randomisation will be performed by the pharmacy of the UMC Utrecht using computer-generated randomisation at a ratio of 1:1 into two treatment groups. The researchers and participants will not be informed about the randomisation outcome until the trial is completed, and the database is locked.

#### Study medication

The study medication (Utrogestan) 300 mg or placebo 300 mg, vaginal capsules (soft), are provided by Besins Healthcare. The capsules are packed in white high-density polyethylene bottles of 15 capsules, with a white polypropylene child-resistant screw cap. The capsules are oblong yellowish, soft gelatin, approximately 2.5 cm × 0.8 cm, containing a whitish oily suspension. The excipient is soyabean lecithin (3 mg per capsule) and sunflower oil (refined). The capsule shell excipients are gelatin, glycerol, titanium dioxide and water. The manufacture and control of the placebo are essentially the same as for the active, but without progesterone drug substance and with appropriately compensating quantities of some excipients.

Utrogestan is a commonly administered medicine in fertility treatment worldwide and is registered in the European Union (EU) for use in assisted reproductive techniques (ART) cycles (Marketing Authorisation number 60954, 1 June 2021). The overall toxicological and safety profile of the progesterone products is well established. Potential side effects (vaginal discharge, mild stomachache, headache or fatigue) are well known, and the use of micronised progesterone is proven to be safe during (early) pregnancy for both mother and child.

#### Accountability

The pharmacy of the UMC Utrecht will keep drug accountability according to local standard operating procedures and national regulation drug accountability. Compliance to therapy is measured via online diaries sent to participants. The pharmacy of the UMC Utrecht will distribute the study medication to the participants’ home address. Upon approval by Besins Healthcare, undistributed study medication will be destructed at the UMC Utrecht. Unused medication (delivered to participants) can be handed in at any local pharmacy, where the medication will be destructed. A summary of the product characteristics (SmPC) of Utrogestan 300 mg is provided as online supplemental file 1.

#### Concomitant therapy

Concomitant medications are allowed as far as they are safe for usage in a subsequent pregnancy. Progesterone may affect the working mechanism of other medicines, and other medicines may affect the metabolism of progesterone. Therefore, the prescribing physician will ensure that the possibilities of drug interaction will be inventoried and appropriate measures will be taken if co-medications as mentioned in the SmPC are used.

#### Study timeline

**Figure 1:**
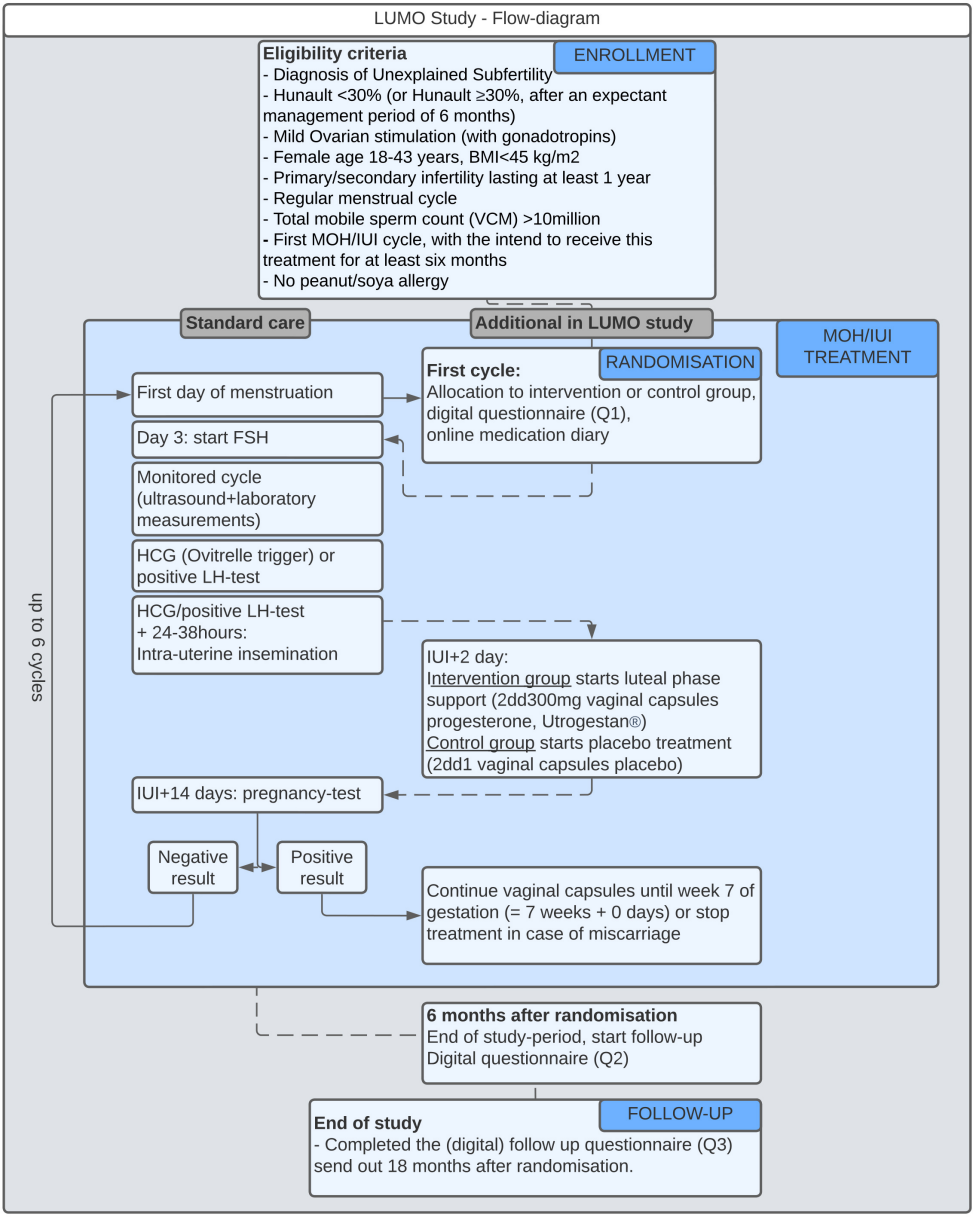
Overview of the main procedures that participants will undergo during standard MOH-IUI treatment and additional actions required for the LUMO trial. BMI, body mass index; FSH, follicle-stimulating hormone; HCG, human chorionic gonadotropin; IUI, intrauterine insemination; LH, luteinising hormone; MOH, mild ovarian hyperstimulation; VCM, total motile sperm count.

MOH-IUI treatment is conducted according to local treatment protocols. Women assigned to the intervention group will start LPS, applying a daily dosage of 2dd 300 mg Utrogestan in vaginal capsules on the second day after IUI (IUI+2 days). Women assigned to the control group will daily start medication of 2dd 300 mg vaginal capsules with placebo, also on the second day after the IUI. Treatment is continued until the onset of menstruation, a negative pregnancy test, miscarriage or until 7 weeks of gestation. Figure 1 presents an overview of the procedures that participants will undergo during standard MOH-IUI treatment and additional actions required for the LUMO study.

The study period is 6 months. Study medication is used after all MOH-IUI treatment cycles within this period. The follow-up period includes the subsequent 12 months after the end of the study period. During the study period and follow-up period, participants do not need to visit the hospital more frequently. There are no additional examinations or blood tests in addition to standard care. No invasive procedures need to be performed. Study participation ends 18 months after randomisation.

A total of three questionnaires will be sent during the study period. At randomisation, questionnaire 1 (Q1) will be sent that contains questions regarding quality of life (FertiQoL). 6 months after randomisation, questionnaire 2 (Q2) will be sent. This questionnaire contains questions regarding quality of life (FertiQoL), compliance to therapy and side effects. Questionnaire 3 (Q3) will be sent 18 months after randomisation. In the case of an ongoing pregnancy within the study period, questionnaire Q3A is sent that contains questions about the pregnancy (complications), delivery and perinatal outcomes. Questionnaire Q3B is sent in case a couple did not achieve an ongoing pregnancy during the study period. This questionnaire contains questions regarding information on the use of additional ART. After every MOH-IUI cycle during the study period, the online medication diary keeps track of compliance to therapy and side effects.

#### Withdrawal

Participants can leave the study at any time for any reason if they wish to do so, without any consequences. The investigator can decide to withdraw a participant from the study for urgent medical reasons. In the case of withdrawal of consent, outcomes will be imputed using all information obtained before withdrawal of consent.

#### Unblinding

In case of a medical emergency requiring unblinding, the pharmacy of the UMC Utrecht will provide a 24/7 backup service. The decision to unblind is at the discretion of the local investigator. If applicable, unblinding information will be accessible to people who need to be involved in the safety reporting to the European Medicines Agency, Data Safety Monitoring Boards (DSMB) or people performing ongoing safety evaluations during the clinical trial.

### Outcomes

The primary outcome is cumulative pregnancy achieved within 6 months after randomisation, leading to live birth rate. Secondary outcomes include (1) progression to in vitro fertilisation (IVF); (2) multiple pregnancy rate; (3) time to pregnancy leading to live birth; (4) pregnancy complications (ie, hypertensive pregnancy disorders, blood loss during early pregnancy, preterm delivery); (5) perinatal outcomes (ie, stillbirth, birth weight, gestational age at delivery, congenital anomalies); (6) delivery outcomes (ie, type of delivery: spontaneous/induced, vaginal/caesarean, blood loss >1 L during delivery, manual removal of placenta); (7) side effects (incidence of common side effects: headache, vaginal discharge/itching, stomach ache, fatigue, nausea and uncommon/other side effects); (8) compliance to therapy; (9) quality of life; (10) number of adverse events (AEs) and serious adverse events (SAEs); and (11) cost-effectivity.

### Sample size

The predicted 6-month ongoing pregnancy leading to a live birth rate for regular MOH-IUI treatment is 30% based on Dutch trials that compare MOH-IUI treatment with expectant management or IVF treatment for couples with unexplained subfertility.^20 21^ Based on the meta-analysis performed by Green *et al* and our own updated meta-analysis, the expected 6-month ongoing pregnancy leading to a live birth rate in the LPS group is approximately 39% (Green review; overall live birth rate RR (relative risk) 1.76 (95% CI 1.29 to 2.40); Casarramona updated review; cumulative live birth rate (up to six cycles) RR 1.49 (95% CI 1.17 to 1.90)).^16 17^ The a priori sample size calculation showed that 479 couples in each treatment arm would provide 80% power, with confidence level 95% to detect the predicted difference of 9% in cumulative live birth rate. To correct for potential dropout (±5%), the aim is to include a total of 1008 patients (504 patients in both groups).

### Data collection and data analysis

Data will be collected in a web-based registration system.^22^ Database cleaning will be carried out by internal consistency checks and identification of database entries outside expected ranges. All statistical analysis will be done according to the intention-to-treat principle. Primary and secondary outcomes will be compared between the treatment arms and expressed as relative risk with 95% CI. Results will be reported per-patient and per-(first) cycle. Time to pregnancy leading to a live birth will be visually compared in a Kaplan-Meier plot and formally compared using log-rank analysis. We will perform a subgroup analysis in which we will assess the effect of potential effect modifiers, like age, type of subfertility (primary or secondary) and duration of subfertility on the effectiveness of progesterone treatment. Attempts will be made to complete missing data (eg, by inquiring at the local sites). If data are still considered missing, cases will be listwise excluded. SPSS (IBM SPSS STatistics V29), RStudio and Excel will be used to perform the statistical analysis.^23^ A p value of <0.05 will be considered statistically significant.

Additionally, a cost-effectiveness analysis and a cost-utility analysis will be performed from a societal and healthcare perspective according to Dutch guidelines with a time horizon of 12 months.

Included cost categories are (1) healthcare costs (MOH-IUI treatment, progesterone capsules, costs of additional hospital visits and IVF treatment in case couples do not become pregnant) and (2) societal costs (mainly related to productivity costs due to treatment and hospital visits).

### Monitoring

Monitoring will be performed in compliance with Good Clinical Practice and other rules and regulations to achieve high-quality research and secure patient safety. Monitoring will be coordinated by the NVOG Consortium and will be executed by a qualified independent monitor in each participating site every year. The study-specific monitoring plan is submitted online within the Central Committee on Research Involving Human Subjects (CCMO) dossier (CTIS) and is stored in the trial master file and digital Research folder. Although the study is ranked as a low-risk study, there will be a DSMB appointed during this trial. The DSMB will monitor SAEs and will advise on continuation or termination of the study based on the interim analysis. The interim analysis is planned at a total of 480 recruited couples.

### Patient and public involvement

The patient federation Freya was invited early in the process to discuss the trial design of the LUMO study. They expressed their support as they emphasised the need for a scientific study to clarify the potential effectiveness of LPS in MOH-IUI treatment. Freya additionally contributed to the study setup by helping develop a survey to consider the opinions of couples with fertility issues on this trial design. The researchers and Freya had multiple consultations during the trial setup phase to discuss the feasibility of the project and formulation of patient-related outcome measures. Freya was also involved in writing the PIF and questionnaires to collect data on compliance/side effects, cost-effectivity and pregnancy outcomes. During the trial, the researchers and Freya will be in contact throughout the entire study period. Freya will share information on the LUMO study (as presented to the ethical committee) and refer to the webpage of the trial (https://www.zorgevaluatienederland.nl/evaluations/lumo) via social media and their own website (https://www.freya.nl).

## Ethics and dissemination

The study is conducted according to the principles of the Declaration of Helsinki (adopted by the 18th WMA General Assembly, Helsinki, Finland, June 1964, and amended by the 64th WMA General Assembly, Fortaleza, Brazil, October 2013) and in accordance with the Medical Research Involving Human Subjects Act (WMO). The study was approved by the CCMO on 30 January 2023. The study is registered in the EU CTIS trial register (2022-501534-33-00), the Dutch trial registry (registration number: LTR 24508), ClinicalTrials.gov (NCT05080569) and the WHO registry (universal trial number: U1111-1280-9461). All participating sites have approval of local Board of Directors to participate in the LUMO study. Amendments will be communicated with the relevant parties. The trial results will be published in peer-reviewed journals and presented at international congresses. Participants will be informed about the results after the completion of the study. An informed consent form will be signed by all participants. A blank copy of the patient information and informed consent file (PIF-IC) of the LUMO trial is provided as online supplemental file 1.

## Discussion

Progesterone luteal support after IVF or intracytoplasmic sperm injection treatment is associated with increased pregnancy and live birth rates and is therefore applied worldwide.^24^ However, there is no consensus on the use of LPS after MOH-IUI treatment for unexplained subfertility. Multiple previous studies have reported a possible beneficial effect of LPS after MOH-IUI in terms of clinical pregnancy. However, the quality of these studies is considered to be low to moderate and study conclusions should therefore be considered with caution.^16 17^ Additionally, due to variations in the administration route, dose and form of LPS, and duration of LPS treatment, the heterogeneity of these studies is high. This underlines the need for a large RCT.

To target a more homogeneous population, only patients starting with MOH-IUI with FSH-stimulated cycles are included in the LUMO trial. According to the Dutch NVOG guidelines, the use of CC to stimulate the ovaries during MOH-IUI treatment in couples with unexplained subfertility is not recommended as it is not considered to be (cost)effective.^25 26^ Additionally, in the meta-analysis by Casarramona *et al*, the observed effect of LPS is more effective in FSH-stimulated cycles than in cycles using CC for stimulation. This may be due to the competitive binding of hypothalamic oestrogen receptors by CC that would mitigate the negative feedback exerted by elevated estradiol levels.^17^

For the present study, two times per day 300 mg vaginal capsules were selected as dosage and administration route. The vaginal route of administration in the form of vaginal gelatin capsules offers a reliable steady-state progesterone (P4) plasma concentration without the high peak levels observed after oral administration. A P4 dosage of 300 mg twice per day in vaginal gelatin capsules creates steady-state levels of approximately 40 nMol/L, which is in the optimal desired range of midluteal P4 levels.^27^,^31^ The oral administration of P4 (tablets) is not recommended due to a first-pass effect resulting in poor absorption when P4 is not micronised.^29^ In medically assisted reproduction in the Netherlands, the use of vaginal progesterone is by far the most applied luteal support route without safety risks as far as current evidence can establish. To ensure sufficient exposure of the endometrium to progesterone and as progesterone levels in the mid-luteal phase below a level of 25 nMol/L are related to poorer pregnancy rates, the start of exogenous progesterone support is best timed before the declining P4 levels reach such levels.^32^ Starting the medication on day 4 after the hCG trigger (IUI+2 days) will help in slowing down the P4 decline and will ensure maintaining P4 levels at the appropriate levels of between 35 and 75 nmol/l.^13 32^ If a pregnancy is established, the luteal–placental shift occurs between week 6 and 8 of gestation. Progesterone production is then taken over by the placenta, and it will no longer be dependent on the corpus luteum.^24^ Therefore, in the case of pregnancy, participants will stop taking the capsules at 7 weeks of gestation. The placebo treatment arm was chosen to ensure the highest level of evidence and maximal adherence to the assigned medication. Compliance with the study medication will be assessed via self-reported outcomes (online medication diaries), rather than through objective endpoints (ie, measuring serum levels of progesterone or returning unused study medication). Previous studies within the consortium demonstrated challenges when return of medication was required, probably due to administrative workload for both research teams and participants. In real-world practice, patients occasionally forget to take their medication. Therefore, outcomes based on strict medication compliance could also have led to results which are less applicable to the general population.

Yearly, approximately 9500 couples enter a MOH-IUI treatment in the Netherlands. Therefore, it seems feasible to achieve the intended sample size inclusion aim. Participants may object to study participation due to the possible assignment to the placebo arm (with a possible nil effect on pregnancy prospects), as well as potential side effects. However, a questionnaire among potential participants during the initiation phase demonstrated that, despite the trial design, they were willing to participate in the LUMO trial.

If the addition of LPS leads to increased success rate of MOH-IUI treatment, we expect it to be a cost-effective addition, and it should therefore be recommended after each MOH-IUI cycle for the fact that progesterone costs are minimal for the proposed dosage for the favourable safety profile and its wide availability. The total number of IUI cycles may be reduced, and fewer couples may need to proceed to invasive and expensive IVF treatments to fulfil their child wish. This could lead to an essential efficacy gain not only from a healthcare perspective, but also from a much larger societal perspective.

In conclusion, the LUMO study is a large, multicentre, double-blind, randomised-controlled trial that aims to determine whether the addition of progesterone LPS after MOH-IUI improves the chance of pregnancy leading to live birth in patients with unexplained subfertility and whether this intervention will be cost-effective.

### Study status

Participants are currently being recruited and enrolled (start date: 6 March 2023). Estimated completion of the study is 1 July 2027.

## Supplementary material

10.1136/bmjopen-2025-111872 online supplemental file 1
