# Correction: Does luteal phase support in MOH-IUI treatment improve cumulative live birth rates in couples with unexplained subfertility? Study protocol of the LUMO study: a multicentre, randomised, double-blind, controlled trial with cost-effectiveness analysis

**DOI:** 10.1136/bmjopen-2025-111872corr1

**Published:** 2026-02-12

Preesman E, Drechsel K, Crommelin H*, et al*. Does luteal phase support in MOH-IUI treatment improve cumulative live birth rates in couples with unexplained subfertility? Study protocol of the LUMO study: a centre, randomised, double-blind, controlled trial with cost-effectiveness analysis. *BMJ Open* 2025;**15:**e111872. doi: 10.1136/bmjopen-2025-111872.

This article has been updated since it was first published. The study design in the title was incorrectly described as “a centre, randomised, double-blind, controlled trial”.

