# Supplementary material for: Does luteal phase support in MOH-IUI treatment improve cumulative live birth rates in couples with unexplained subfertility? Study protocol of the LUMO study: a multicentre, randomised, double-blind, controlled trial with cost-effectiveness analysis
Source: BMJ Open. 2025 Nov 19;15(11):e111872. doi: 10.1136/bmjopen-2025-111872 (PMC12636955; doi:10.1136/bmjopen-2025-111872)
Supplement: online supplemental file 1 [file bmjopen-15-11-s001.docx]

**Subject information for participation in medical research**

**Study title**

"Does the addition of progesterone to MOH-IUI treatment (mild ovarian stimulation with hormones combined with insemination of processed semen) lead to a higher chance of pregnancy?” **(the LUMO study)**

*Officiële titel:* *Effectiviteit van luteale-fase ondersteuning met progesteron na intra-uteriene inseminatie in een mild gestimuleerde cyclus.*

**Introduction**

Dear Madam and partner,

With this letter, we would like to ask you to participate in a medical study. Participation is voluntary. You have received this letter because you have visited the gynaecologist or fertility doctor at the hospital because you did not become pregnant after trying for quite some time. After the infertility screening, no clear explanation was found for the failure to conceive and you are eligible for MOH-IUI treatment. This is a treatment in which your cycle is stimulated using low dosage of FSH hormone ("mild ovarian stimulation," MOH) and your partner’s sperm cells, after a wash procedure, are introduced into the uterus ("intrauterine insemination," IUI). You can read about the medical study in this information sheet, what it means for you, and what the pros and cons are. It is a lot of information. Can you please read the information and decide if you want to participate? If you want to participate, complete the form in Appendix E.

**Ask your questions**

You can take your decision based on the information in this information sheet. We also suggest that you do this:

- Ask your questions to the investigator who gave you this information.

- Talk to your partner, family or friends about this study.

- Read the information on [www.rijksoverheid.nl/mensenonderzoek](http://www.rijksoverheid.nl/mensenonderzoek).

1. **General information**

The UMC Utrecht has set up this study. Below, we always call the UMC Utrecht the 'sponsor'. Investigators, these can be doctors/researchers/research nurses, conduct the study in different hospitals. A total of 1008 women will participate in this study. These women will visit 1 of the 29 hospitals cooperating in the research consortium of the NVOG (the Dutch Society of Obstetrics and Gynaecology). The Central Committee on Human Research (CCMO) has approved this research. The research is funded by ZonMW (independent funder) and conducted by UMC Utrecht in collaboration with the NVOG consortium 2.0. A part of the study is paid for by a grant from the company Besins Healthcare, manufacturer of Utrogestan. Besins Healthcare has no input into the design or conduct of the study.

1. **What is the purpose of the study?**

In this study, we look at whether the addition of progesterone to MOH-IUI treatment leads to a higher chance of pregnancy. We compare the effect of progesterone with the effect of a placebo. A placebo is a drug with no active ingredient. If progesterone increases the likelihood of pregnancy, women will need fewer MOH-IUI treatments and there are also fewer couples who will later need to use IVF/ICSI treatments ("in vitro fertilization/ intra-cytoplasmic sperm injection (IVF/ICSI), also known as test tube fertilization).

1. **What is the background of the study?**

The stimulation of egg ripening and inducing ovulation with hormones, as happens during MOH-IUI treatment, can cause a shortage of progesterone after ovulation. The uterus then may not be optimally prepared for embryo implantation. We know that the addition of progesterone supplementation in other fertility treatments (IVF/ICSI) leads to a higher chance of pregnancy after treatment. Existing studies on the use of progesterone in MOH-IUI are still of insufficient quality, therefore we want to study the effect of progesterone in a large study.

1. **What happens during the study?**

*How long will the study take?*

Are you participating in the study? Study participation will take about 18 months in total (a year and a half). During the first 6 months, you will use the study medication in every MOH-IUI treatment cycle you undergo. We would like to ask you some questions in the following year. We will do this with an online questionnaire, which will be sent to you about 18 months after the start of the study.

*Step 1: are you eligible to participate?*

First, we want to know if you are eligible to participate. Therefore, the investigator will check the following:

- You are between 18 and 43 years old.
- The infertility examination has found no obvious reasons for the absence of pregnancy.
- You will start MOH-IUI treatment, during which your cycle will be stimulated with low doses of FSH hormones.
- You have no allergies to soy or peanuts.

*Step 2: draw for progesterone or no progesterone*

For this study, there will be 2 study groups:

- Group 1. The women in this group will apply vaginal capsules with progesterone (Utrogestan with 300mg progesterone per capsule) after insemination.
- Group 2. The women in this group will apply vaginal capsules with placebo (with no active ingredient) after insemination.

Both groups insert the vaginal capsules twice a day (morning and evening) starting on day 2 after insemination. You will continue using the capsules until your menstruation starts, you have a negative pregnancy test (you take a pregnancy test 14 days after the insemination), or until week 7 of pregnancy (7 weeks and 0 days, this is 5 weeks after the insemination date). If you unfortunately have a miscarriage, you stop using the capsules. You will use the capsules during every MOH-IUI treatment you undergo within 6 months after the onset of your study participation.

Drawing will determine which group you will be placed in. You and the investigator do not know which group you are in. But if it is important for your health, we can look this up.

You will be given the same vaginal capsules each time for the entire 6-month period. It is not possible to find out which group you are in during the study. Not even if you unexpectedly have a miscarriage. This is because it is important for the quality of the study that neither you nor the investigator know which medication you are receiving for the entire study period. When the study is finished (expected after about 4-5 years), you may be told which group you were in.

*Step 3: study and measurements*

If you participate in this study, you do not need to visit the hospital more often than normal in this treatment, even if you become pregnant during the study. You will use the study medication as prescribed. You will keep a record of when you have used a vaginal capsule. In addition, you will report any symptoms in an (online) diary.

At the beginning of the study, after 6 months and after 18 months, you will be contacted by e-mail to complete an online questionnaire. These questionnaires will include questions about your quality of life. These questions are part of a standardized questionnaire. Therefore, the investigators cannot remove certain questions from the questionnaire. It is possible that not all questions fully reflect your current situation. Nevertheless, we kindly ask you to answer all questions. After 6 months, we will also ask how you experienced the treatments, whether you used all the prescribed medication and whether you experienced any side effects from the medication. If you become pregnant in the first 6 months after the draw, there will be questions on the course of the pregnancy and the health of your baby within the questionnaire after 18 months. If you did not become pregnant in the first 6 months after the draw, you will be asked if you had any fertility treatments and/or became pregnant in the year after study participation.

A summary of what is expected of you during the study is included in Appendix C.

*What is the difference with standard care?*

The first part of the MOH-IUI treatment proceeds as normal care. After the insemination, you will use the study medication (vaginal capsules) twice a day for up to 5 weeks. You keep track of when you insert the vaginal capsules and whether you experience any side effects. You do not need to visit the hospital more often. You will receive 3 invitations to complete a questionnaire.

1. **What agreements do we make with you?**

We want the study to go well. That is why we want to make the following agreements with you:

- You take the study medication in the way the investigator explained to you. You keep track of when you inserted the study medication and whether you experience any side effects.
- You do not participate in any other medical research during this study.
- You should contact the investigator in these situations:
  - You want to start taking other medication. Also, if these are homeopathic remedies, natural remedies, vitamins or over-the-counter medicines. You do not need to contact us if you start using folic acid or multivitamins for pregnancy.
  - You are hospitalised or get treatment in a hospital.
  - You suddenly have problems with your health.
  - You no longer want to participate in the study.
  - Your telephone number, address or email address changes.

1. **What side effects, adverse effects or discomforts could you experience?**

The study medication to be investigated may cause side effects, although these occur in a fairly low rate.

The following side effects are common:

- Vaginal discharge

- Vaginal itching or burning sensation

- Nausea, abdominal pain or cramps, bloated abdomen, constipation

- Sore or sensitive breasts

- Blood loss from the vagina, beyond normal monthly bleeding

- Fatigue, drowsiness

- Headache

More information about Utrogestan (vaginal capsules containing progesterone) is in the information leaflet, see Appendix D.

Please note! Both groups will receive the information leaflet about Utrogestan. The investigators (and yourself) do not know which group you are in.

The study medication can also have side effects that we do not know about at the moment. The chance of this is minimal, since there is already a lot of experience with the capsules in other fertility treatments. Please contact the investigator if you experience any serious side effects that are not listed, serious or otherwise. This includes side effects not listed above or in the information leaflet.

There is always a small chance of birth defects in any pregnancy. These are not covered by the subject-insurance coverage for this study. However, no increased risk of anomalies is observed in women who already used vaginal capsules of progesterone in early pregnancy, such as in the practice of IVF treatment.

**Placebo**

The study-group that receives a placebo (vaginal capsules with no active ingredient) may also suffer of side effects. The main ones are vaginal discharge, itching or burning.

1. **What are the pros and cons if you participate in the study?**

Participating in the study can have pros and cons. We will list them below. Think about this carefully and talk to other people about it.

Participating in the study can have these **pros**:

- Because it is a research study, it is not clear whether you will personally benefit from participating in this study. The chance of pregnancy after MOH-IUI treatment may increase thanks to progesterone, but this is not certain. Previous studies and experience with progesterone treatment in other fertility treatments did not show any increased risk of miscarriage or multiple pregnancy. The drug is safe to use during (early) pregnancy.

- The data from this study may be of longer-term benefit to women in your situation.

Participating in the study can have these **cons**:

- - You may experience side effects or adverse effects as described in section 6.
  - You have to comply with the study agreements.
  - You will need to complete a questionnaire 3 times. This takes about 10-25 minutes each time. The questionnaires will include questions on quality of life and you may find these questions confronting.

*You do not wish to participate in the study?*

It is up to you to decide whether you wish to participate in the study. Participation is voluntary. If you do not wish to participle, you will receive the standard MOH-IUI treatment that you would have received otherwise. You do not have to say why you do not want to participate. If you do participate, you can always change your mind and stop anyway. Even during the study. Progesterone treatment is not part of standard care and will not be part of standard care until the results of this study are known. Therefore, you cannot choose to receive progesterone yourself.

1. **When does the study end?**

The investigator will let you know if there is any new information about the study that is important to you. The investigator will then ask you if you want to continue to participate.

In these situations, the study will stop for you:

- You used the study medication in MOH-IUI treatments up to 6 months after the draw and completed the last online questionnaire, 18 months after the draw.
- You want to stop participating in the study yourself. You can stop at any time. Report this to the investigator immediately. You do not have to explain why you want to stop.
- The investigator thinks it is better for you to stop. The investigator will still invite you for a follow-up check.
- One of the following authorities decides that the study should stop:
  - the government, or
  - the Medical Ethics Review Committee assessing the study

*What happens if you stop participating in the study?*

You will then receive the usual MOH-IUI treatment again, without additional medication after insemination. The investigators use the data collected up to the time of stopping.

The entire study ends when all the participants (1008 women) have finished.

1. **What happens after the study has ended?**

*Can you continue taking the study medication?*

You will not be able to use the vaginal capsules after completion of the study. Even if you were to start a new MOH-IUI treatment 6 months after the draw.

*Will you get the results of the study?*

After the study has ended and as soon as the results are ready for publication, the investigator will inform you about the most important results of the study. The investigator may also tell you what group you were in. Do you prefer not to know? Please indicate this on the consent form (Appendix E). He/she will not tell you in that case.

1. **What will be done with your data?**

Are you participating in the study? Then you also give your consent to collect, use and store your data.

*What data do we store?*

We store these data:

- your name

- your gender

- your address

- your date of birth

- your email address

- information about your health

- (medical) information that we collect during the study

The outcome of your potential pregnancy is also important, therefore, we may want to request additional information about your pregnancy, delivery and child(ren). This will include any pregnancy complications, course of labour and postnatal period, gestational age at birth, height, weight, and any health problems in your child(ren) that occurred in the first year after birth.

Because of the fertility treatment, we also collect data from your partner. We store the following data of your partner:

- month and year of birth

- data about his health, such as height and weight

- (medical) data we collect during the study that are about your partner, such as sperm quality

We ask your partner to consent to the collection of data about his health and that of the (unborn) child by co-signing the consent form.

*Why do we collect, use and store your data?*

We collect, use and store your data to answer the questions of this study. And to be able to publish the results. Your email address will be used to send online questionnaires. Your address information will be shared with the UMC Utrecht pharmacy (Clinical Medicine Research Department; KGO) to send the study medication to your home address.

*How do we protect your privacy?*

To protect your privacy, we give a code to your data. We only put this code on your data. We keep the key to the code in a safe place in the hospital. When we process your data, we always use only that code. Even in reports and publications about the study, nobody will be able to see that it was about you.

*Who can see your data?*

Some people can see your name and other personal information without a code. These are people checking whether the investigators are carrying out the study properly and reliably. These persons can access your data:

- Members of the committee that keeps an eye on the safety of the study.
- An auditor who is hired by the sponsor
- National and supervisory authorities. For example, the European Medicines Agency (EMA). Furthermore, it is necessary that your name and address details are shared with the pharmacy of the UMC Utrecht (Department of Clinical Drug Research; KGO) in Utrecht, so they can send you the study medication.

These people will keep your information confidential. We ask you to give permission for this access. The Health and Youth Inspectorate (IGJ) can access your personal information without your permission.

*For how long do we store your data?*

We store your data in the hospital for 25 years. And for 25 years with the sponsor (the UMC Utrecht).

*Can we use your data for other research?*

Your collected data may also be important for other medical research on the field of IUI treatments with progesterone supplementation. For this purpose, your data will be stored in the hospital for 25 years. Please indicate in the consent form whether you agree with this. Do you not want to give your consent? Then you can still participate in this study. You will get the same healthcare.

*What happens if there are coincidental findings?*

It is possible that during the study we discover something that is not directly relevant to the study but is important to your health. In that case, the investigator will contact your general practitioner or treating doctor. You will then discuss what needs to be done with general practitioner or treating doctor. With the form, you give consent to inform your doctor or specialist.

*Can you take back your consent for the use of your data?*

You can take back your consent for the use of your data at any time. Report this to the investigator immediately. This applies both to the use in this study and to the use in other medical research. But please note: if you take back your consent, and the investigators have already collected data for research, they are still allowed to use this information.

*Do you want to know more about your privacy?*

- Do you want to know more about your rights when processing personal data? Visit [www.autoriteitpersoonsgegevens.nl](http://www.autoriteitpersoonsgegevens.nl).
- Do you have questions about your rights? Or do you have a complaint about the processing of your personal data? Please contact the person who is responsible for processing your personal data. For the present, this is:
  - [Institution name] and the UMC Utrecht, See Appendix A for contact details, and website.
- If you have any complaints about the processing of your personal data, we recommend that you first discuss them with the research team. You can also contact the Data Protection Officer of [the institution], or the UMC Utrecht. Or you can submit a complaint to the Dutch Data Protection Authority.

*Where can you find more information about the study?*

You can find more information about the study on the following website: [www.zorgevaluatienederland.nl/LUMO](http://www.zorgevaluatienederland.nl/LUMO). After the study, the website may show a summary of the results of this study.

1. **Will you receive compensation if you participate in the study?**

The study medication for the study will not cost you anything. Neither will you get any compensation if you participate in this study.

1. **Are you insured during the study?**

Insurance has been taken out for everyone who takes part in this study. The insurance pays for damage caused by the study. But not for all damage. You can find more information about this insurance and any exceptions in **Appendix B**. It also says who you can report damage to.

1. **We will inform your general practitioner and pharmacist in your hospital**

The investigator will send your general practitioner and pharmacist a letter to let them know that you are participating in the study. This is for your own safety. We may contact your general practitioner, for example, about your medical history.

1. **Do you have any questions?**

You can ask questions about the study to the research team at the hospital where you are being treated. An informative video about the study can be found at www.zorgevaluatienederland.nl/LUMO. You can also scan the QR code in Appendix A with your phone or tablet to get to this web page. Do you have a complaint? Discuss it with the investigator or the doctor who is treating you. If you prefer not to do so, please visit the complaints officer of your hospital. Appendix A tells you where to find this.

1. **How do you give consent for the study?**

You can first think carefully about this study. Then you tell the investigator if you understand the information and if you want to participate or not. If you want to participate, fill in the consent form that you can find with this information sheet. You and the investigator will both get a signed version of this consent form.

Thank you for your attention.

Yours sincerely,

On behalf of the entire research team

Dr. S.L. Broer, Gynaecologist Reproductive Medicine, UMC Utrecht, the Netherlands

Principal investigator LUMO study

1. **Appendices to this information**

A. Contact details

B. Information about the insurance

C. Schedule of study interventions

D. Information leaflet Utrogestan 300mg, vaginal capsules

E. Consent form(s)

**Appendix A: contact details for [name of participating centre]**

**Investigators:**

[for principal investigator of centre: name, contact details and accessibility]

< if applicable>

[Study nurse/study doctor/nurse specialist]:

**Complaints:**

[service or person with contact details and accessibility]

**Data Protection Officer of the institution:**

[email]

For more information about your rights: [www.autoriteitpersoonsgegevens.nl](http://www.autoriteitpersoonsgegevens.nl).

**Investigators UMC Utrecht:**

Dr. S.L. (Simone) Broer, principal investigator

Drs. K.C.E. (Katja) Drechsel, trial coordinator

Drs. E.E. (Emma) Preesman, medical doctor-investigator

Email: lumo@umcutrecht.nl

Tel: 06-25710994

Complaints UMC Utrecht:

Address: Klachtenbemiddeling UMC Utrecht, Huispost Q05.4.310, Replynumber 8419, Postbox 85500, 3508 GA Utrecht

Tel: 088-7556208

UMC Utrecht Data Protection Officer:

privacy@umcutrecht.nl


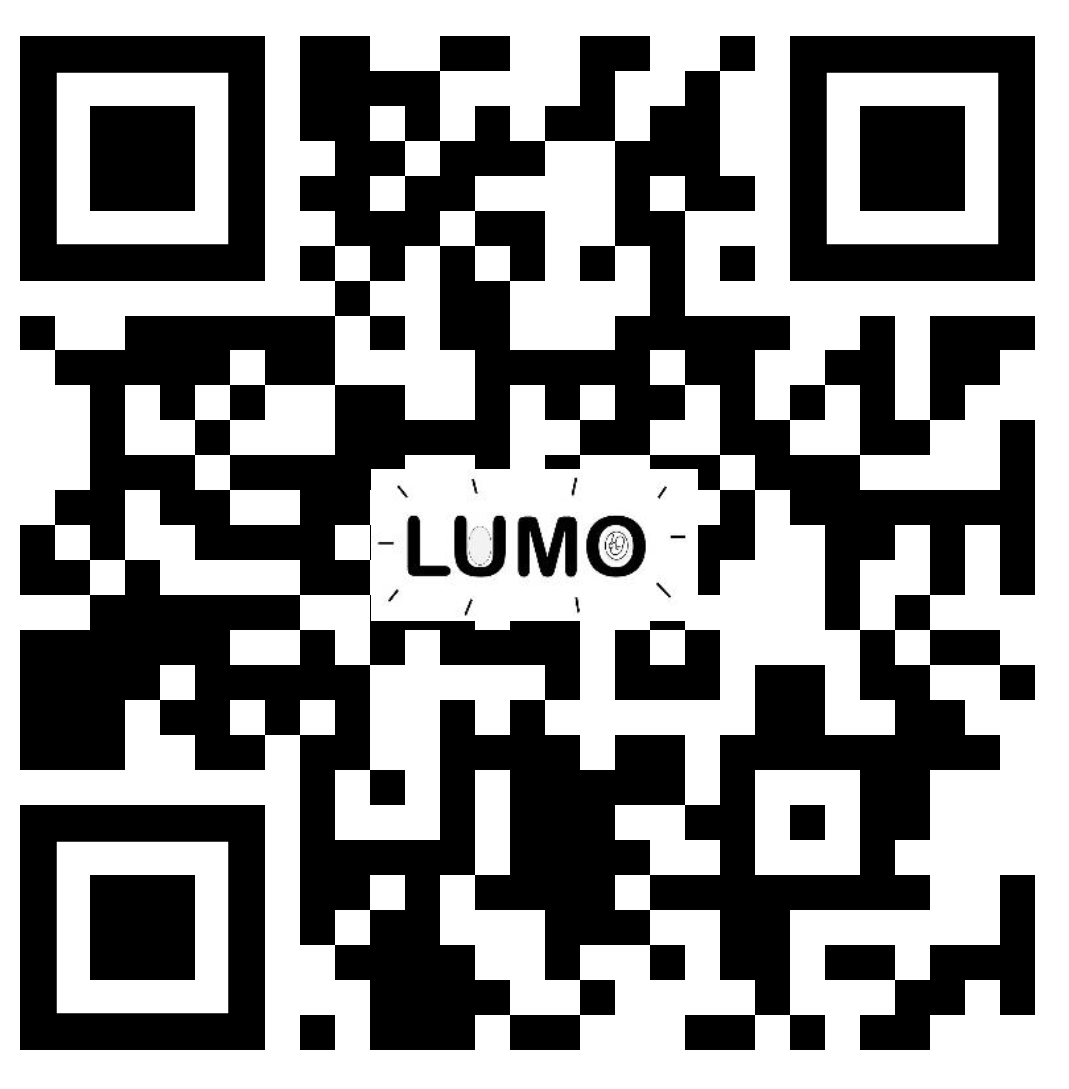

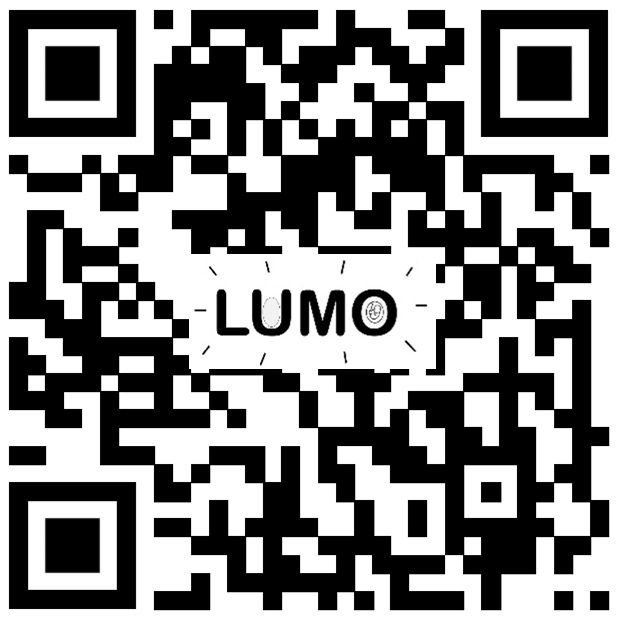
**Website LUMO study:**

For an informative video about the study, go to: www.zorgevaluatienederland.nl/LUMO

Or scan the QR code to the right with your tablet or smartphone to get to the webpage.

**Appendix B: information about the insurance**

The UMC Utrecht has taken out insurance for everyone who takes part in the study. The insurance pays for the damage you have suffered because you participated in the study. This concerns damage you suffer during the study or within 4 years after you participated in the study. You must report damage to the insurer within 4 years.

Have you suffered damage as a result of the study? Please report this to this insurer:

The insurer of the study is:

Name insurer: CNA Insurance Company (Europe) S.A.

Address: Polarisavenue 140, 2132 JX Hoofddorp, Nederland

Telephone number: 0031 (0)23-3036002

Email: claimsnetherlands@cnahardy.com

Policy number: 10463520

The insurance pays a maximum €650,000 per person and €5,000,000 for the entire study (and € 7,500,000 per year for all studies by the same sponsor).

Please note that the insurance does **not** cover the following damage:

- Members of the committee monitoring the safety of the study.
- Damage due to a risk about which we have given you information in this sheet. But this does not apply if the risk turned out to be greater than we previously thought. Or if the risk was very unlikely.
- Damage to your health that would also have happened if you had not taken part in the study.
- Damage that happens because you did not follow directions or instructions or did not follow them properly.
- Damage to the health of your children or grandchildren.
- Damage caused by a treatment method that already exists. Or by research into a treatment method that already exists.

These provisions can be found in the 'Besluit verplichte verzekering bij medisch-wetenschappelijk onderzoek met mensen 2015' ('Medical Research (Human Subjects) Compulsory Insurance Decree 2015'). This decision can be found in the Government Law Gazette (<https://wetten.overheid.nl>).

**Appendix C: Diagram of study interventions**

**
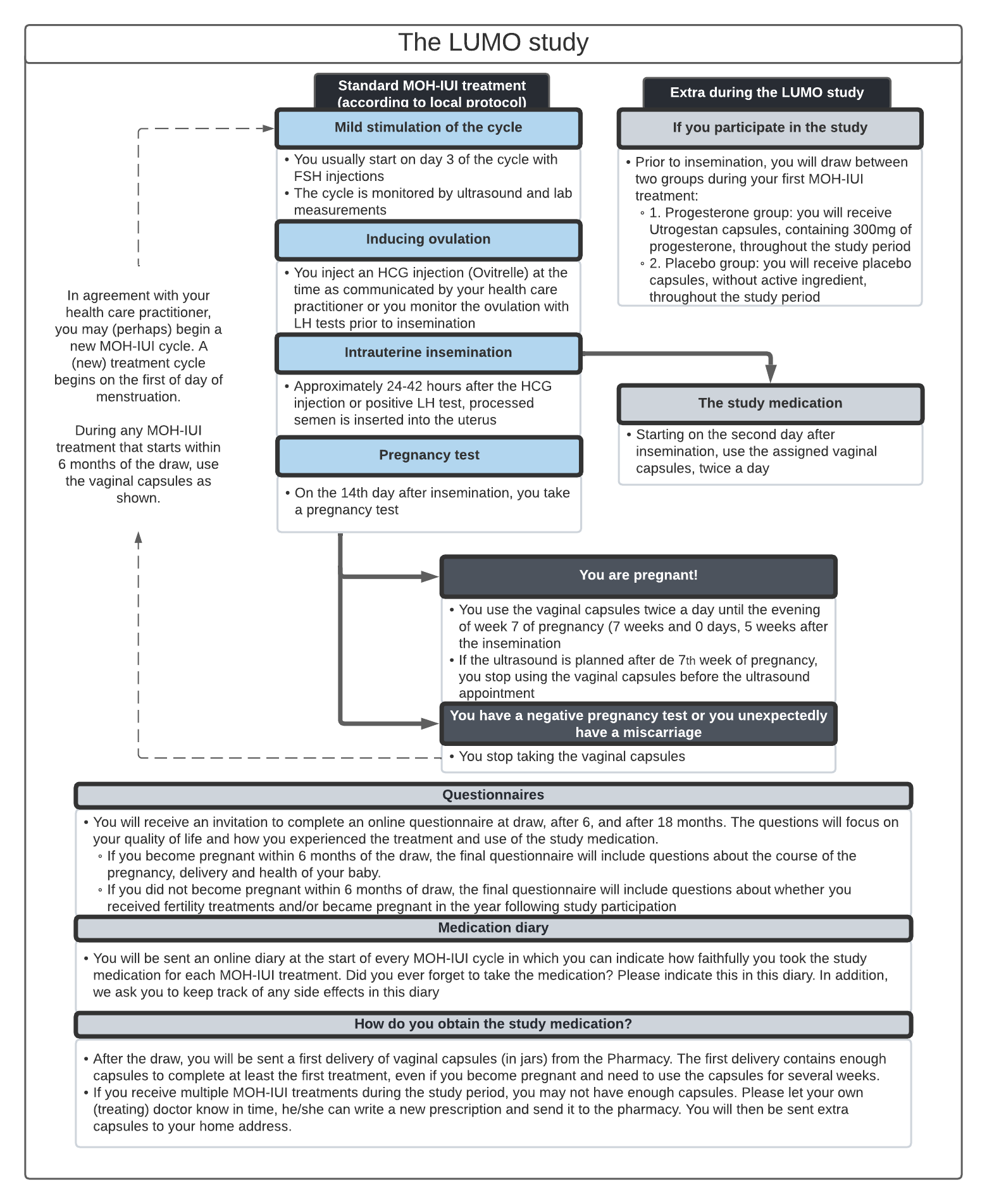
**

**Appendix D – Information leaflet Utrogestan 300mg, vaginal capsules**

**Package leaflet: Information for the user**

Please note: This is the standard leaflet for Utrogestan. This leaflet was not created for the LUMO study. There may be information in the leaflet that does not fully reflect your situation.

Participants have a 50% chance of being randomized into the placebo group. The investigators (and yourself) do not know which group you are in. Everyone receives the leaflet for the progesterone capsules (Utrogestan) with their medication.

**Utrogestan Vaginal 300 mg Vaginal Capsules, soft**

progesterone

**Read all of this leaflet carefully before you start using this medicine because it contains important information for you.**

- Keep this leaflet. You may need to read it again.
- If you have further questions, ask your doctor, pharmacist or nurse.
- This medicine has been prescribed for you only. Do not pass it on to others. It may harm them, even if their signs of illness are the same as yours.
- If you get any side effects, talk to your doctor, pharmacist or nurse. This includes any possible side effects not listed in this leaflet. See section 4.

**What is in this leaflet:**

1. What Utrogestan 300mg and what it is used for

2. What you need to know before you use Utrogestan Vaginal

3. How to use Utrogestan Vaginal

4. Possible side effects

5. How to store Utrogestan Vaginal

6. Contents of the pack and other information

**1. What Utrogestan Vaginal is and what it is used for**

The name of your medicine is Utrogestan Vaginal 300 mg Vaginal Capsules, soft (called Utrogestan Vaginal in this leaflet). Utrogestan Vaginal contains a hormone called progesterone.

**What Utrogestan Vaginal is used for**

Utrogestan Vaginal can be used to support pregnancy during in vitro fertilization (IVF) cycles.

**To support pregnancy**

Utrogestan Vaginal is for women who need extra progesterone while undergoing treatment in an Assisted Reproductive Technology (ART) program.

**2. What you need to know before you use Utrogestan Vaginal**

**Do not use Utrogestan Vaginal**

• if you are allergic (hypersensitive) to soya or peanuts

• if you are allergic to progesterone or any of the other ingredients of this medicine (listed in section 6)

• if you have liver problems

• if you have yellowing of the skin or eyes (jaundice)

• if you have unexplained vaginal bleeding

• if you have breast cancer or genital tract carcinoma

• if you have thrombophlebitis

• if you have or have had blood clots in a vein (thrombosis), such as in the leg (deep vein thrombosis) or the lungs (pulmonary embolism)

• if you have had a cerebral haemorrhage or stroke

• if you have a rare blood disease called porphyria, which is passed down in families (inherited)

• if you are pregnant but your baby has died inside of you (missed abortion).

**Warnings and precautions**

Utrogestan Vaginal should only be used during the first 3 months of pregnancy. Talk to your doctor or pharmacist before using Utrogestan Vaginal. Utrogestan Vaginal is not a contraceptive. If you believe that you have had a miscarriage, you must speak with your doctor, because you must stop using Utrogestan Vaginal. You must contact your doctor if you feel unwell within a few days of taking the medicine.

**Children and adolescents**

Utrogestan Vaginal is not for use in children and adolescents.

**Other medicines and Utrogestan Vaginal**

Tell your doctor or pharmacist if you are taking, have recently taken or might take any other medicines. This includes medicines obtained without a prescription, including herbal medicines. This is because Utrogestan Vaginal can affect the way some other medicines work. Also some other medicines can affect the way Utrogestan Vaginal works.

In particular tell your doctor or pharmacist if you are taking any of the following medicines:

- blood thinners such as coumarins or phenindione
- ciclosporin or tacrolimus (used to reduce the immune response)
- tizanidine (muscle relaxant)
- bromocriptine, which is used in the treatment of pituitary gland-related problems or Parkinson’s disease
- selegiline (for treating Parkinson’s disease)
- diazepam, chlordiazepoxide, alprazolam, oxazepam or lorazepam (for treating anxiety or insomnia)
- tuberculosis medicines (such as rifampicin and rifabutin)
- antibiotics (for example griseofulvin, ampicillin, amoxicillin and tetracyclines), which are used to treat certain infections
- phenytoin, phenobarbital, carbamazepine, eslicarbazepine, oxcarbazepine, primidone/rufinamide, perampanel or topiramate (used for epilepsy)
- herbal medicine products, containing St John´s Wort
- darunavir, nelfinavir, fosamprenavir or lopinavir (used to treat viral infections)
- bosentan (used to treat lung problems)
- fluconazole, itraconazole, voriconazole (for the treatment of fungal infections)
- ketoconazole (used to treat Cushing’s syndrome – when the body produces and excess of cortisol)
- atorvastatin or rosuvastatin (used to control cholesterol)
- aprepitant (used to prevent nausea and vomiting).

If you have recently been given an anaesthetic, such as bupivacaine or

If you have been recently been tested for liver or hormone problems.

**Utrogestan Vaginal with food and drink**

Utrogestan Vaginal should be inserted into the vagina. Food and drink do not affect the treatment.

**Pregnancy, breast-feeding and fertility**

Utrogestan Vaginal will support pregnancy if you are having fertility treatment. For instructions on how to use Utrogestan Vaginal see Section 3 on how to use Utrogestan Vaginal. Do not use Utrogestan Vaginal if you are breast-feeding.

**Driving and using machines**

Utrogestan Vaginal has no or negligible effects on driving or using machinery.

**Utrogestan Vaginal contains soybean lecithin**

If you are allergic to peanut or soya, do not use this medicinal product.

**3. How to use Utrogestan Vaginal**

Always use this medicine exactly as your doctor has told you. Check with your doctor or pharmacist if you are not sure.

**Using this medicine is to assist in getting pregnant**

• Do not take it by mouth. If you accidentally take Utrogestan Vaginal by mouth, it will not harm you, but your chances of becoming pregnant will be reduced.

**Recommended dose**

• The treatment is started no later than the third day after egg retrieval.

• Every day, use 600 mg of Utrogestan Vaginal as instructed by the doctor. Insert one capsule deep into the vagina in the morning and the other at bedtime.

• If laboratory tests confirm that you are pregnant, continue with same dose until the 7th week of pregnancy according to your doctor’s instructions.

**If you use more Utrogestan Vaginal than you should**

If you use too much Utrogestan Vaginal you should talk to your doctor or go to a hospital. Take the medicine pack with you.

The following effects may happen: feeling dizzy or feeling tired.

**If you forget to use Utrogestan Vaginal**

• If you forget a dose, insert it as soon as you remember it. However, if it is nearly time for your next dose, skip the missed dose.

• Do not use a double dose to make up for a forgotten dose.

**If you stop using Utrogestan Vaginal**

Talk to your doctor, pharmacist or nurse before stopping to use this medicine. If you stop using the medicine, it will not help you to get pregnant.

If you have any further questions on the use of this medicine, ask your doctor, pharmacist or nurse.

**4. Possible side effects**

Like all medicines, this medicine can cause side effects, although not everybody gets them. The following side effects may happen with this medicine:

Frequency not known (frequency cannot be estimated from the available data):

- Itching

- Vaginal bleeding

- Oily discharge from the vagina.

Short-term tiredness or dizziness may be experienced within 1 – 3 hours from using the medicine. If you notice side effects other than those mentioned above, tell your doctor.

**Reporting of side effects**

If you get any side effects, talk to your doctor, pharmacist or nurse. This includes any possible side effects not listed in this leaflet. You can also report side effects directly via HPRA Pharmacovigilance Website: www.hpra.ie. By reporting side effects you can help provide more information on the safety of this medicine.

**5. How to store Utrogestan Vaginal**

• Keep this medicine out of the sight and reach of children.

• Do not use this medicine after the expiry date which is stated on the packaging after EXP. The expiry date refers to the last day of that month.

• Once the bottle has been opened, use the capsules within 15 days.

• Store in the original packaging (the bottle).

• Store below 30°C.

• Do not use this medicine if you notice visible changes in the appearance of the medicine.

• Do not throw away any medicines via wastewater or household waste. Ask your pharmacist how to throw away medicines you no longer use. These measures will help to protect the environment.

**6. Contents of the pack and other information**

**What Utrogestan Vaginal contains**

• The active substance is progesterone. One capsule contains 300mg of progesterone.

• The other ingredients in the capsule are refined sunflower oil, soyabean lecithin, gelatine, glycerol (E422), titanium dioxide (E171) and purified water.

**What Utrogestan Vaginal looks like and contents of the pack**

The capsules are oblong yellowish, soft gelatin capsules, that contain a whitish oily suspension. The dimensions of the 300 mg capsule are approximately 2.5 cm x 0.8 cm.

Utrogestan Vaginal are supplied in white high density polyethylene plastic bottles with a child resistant white polypropylene screw cap and a tearable silver coloured seal. Pack size: 1 bottle containing 15 capsules.

**Marketing Authorisation Holder**

The Marketing Authorisation for Utrogestan Vaginal is held by : Besins Healthcare Ireland Limited

16 Pembroke Street Upper

Dublin 2 D02HE63

Ireland

**Manufacturer**

Cyndea Pharma S.L.

Poligono Industrial Emiliano Revilla Sanz Avenida de Agreda, 31

Olvega 42110 (Soria) Spain

**This medicinal product is authorised in the Member States of the EEA under the following names:**

Bulgaria, Estonia, Hungary, Croatia, The Netherlands, Norway, Sweden, Slovenia, Slovakia: Utrogestan Belgium, Ireland, Luxembourg: Utrogestan Vaginal

Finland: Lugesteron

Latvia: Progesterone Besins

**Appendix E: Informed consent form – subject**

Belonging to ''Does the addition of progesterone to MOH-IUI treatment (mild ovarian stimulation with hormones combined with insemination of processed semen) lead to a higher chance of pregnancy?'' (the LUMO study).

- I have read the information sheet about the ‘LUMO-study’. I was able to ask questions. My questions have been answered well enough. I had enough time to decide if I wanted to take part.
- I know that taking part is voluntary. I also know that at any time I can decide not to take part in the study. Or to stop taking part. I do not have to explain why.
- I give the investigator consent to inform my general practitioner and pharmacist that I am taking part in this study.
- I give consent to share my name and address with the pharmacy at UMC Utrecht (Department of Clinical Drug Research; KGO) in Utrecht (for sending the study medication).
- I give consent to share my email address with the researcher at the coordinating center (UMC Utrecht) to send the questionnaires.
- I give consent to give my general practitioner or specialist information about accidental discoveries made during the study that are important for my health.
- I give consent to collect and use my data. The investigators only do this to answer the question of this study.
- I know that some people will be able to see all of my data to review the study. These people are mentioned in this information sheet. I give consent to let them see my data for this review.
- I consent to request our newborn child's records from his/her medical record, given that these records are used in a coded manner for this study.
- Please tick yes or no in the table below.

| I give consent to store my data to use for other research, as stated in the information sheet. | Yes ☐ | No☐ |
| --- | --- | --- |
| I give consent to ask me after this study if I want to participate in a follow-up study. | Yes ☐ | No☐ |
| I give consent to let me know after the study which treatment I received/in which group I was. | Yes ☐ | No☐ |

- I want to take part in this study.

My name is (subject): ……………………………….. Date of birth: __/__/__

Signature: ……………………… Date : __/__/__

-----------------------------------------------------------------------------------------------------------------

I declare that I have fully informed this subject about the study mentioned.

If any information becomes known during the study that could influence the subject's consent, I will let this subject know in good time.

Investigator name (or their representative): ........................

Signature:……………………… Date: __/__/__

-----------------------------------------------------------------------------------------------------------------

<if applicable>

Additional information was given by:

Name:………………………………..

Job title:………………………………

Signature:……………………… Date: __/__/__

-----------------------------------------------------------------------------------------------------------------

*The study subject will receive a complete information sheet, together with a signed version of the consent form.*

**Informed** **consent form – partner of subject**

Belonging to ''Does the addition of progesterone to MOH-IUI treatment (mild ovarian stimulation with hormones combined with insemination of processed semen) lead to a higher chance of pregnancy?'' (the LUMO study).

**Partner**

- I have read the information sheet for the ’LUMO-study’. I was able to ask questions. My questions have been answered well enough. I had enough time to decide if I want my child to take part.
- I know that taking part is voluntary. I also know that I can decide at any time that my child will not take part after all. I do not have to explain why.
- I give consent to collect and use my data. The investigators only do this to answer the question of this study.
- I know that some people will be able to see all of my data to review the study. These people are mentioned in this information sheet. I give consent to let these people see my data for this review.
- I give consent to keep my data for 25 years after this study.
- I give consent to retrieve our newborn child's data from his/her medical record, provided this data is used in a coded manner for this study

Name partner ……………………………… Date of birth partner: __/__/__

Signature: ………………………………… Date: __/__/__

-----------------------------------------------------------------------------------------------------------------

I declare that I have fully informed the partner mentioned above about the said study.

If any information becomes known during the study that could influence the subject of partner’s consent, I will let them know in good time.

Investigator name (or their representative): …………………………

Signature: ……………………… Date: __/__/__

Additional information was given by (if applicable):

Name: ………………………………………..

Job title: ………………………………………

Signature: ………………………………. Date: __/__/__
