# Supplementary material for: Does luteal phase support in MOH-IUI treatment improve cumulative live birth rates in couples with unexplained subfertility? Study protocol of the LUMO study: a multicentre, randomised, double-blind, controlled trial with cost-effectiveness analysis
Source: BMJ Open. 2025 Nov 19;15(11):e111872. doi: 10.1136/bmjopen-2025-111872 (PMC12636955; doi:10.1136/bmjopen-2025-111872)
Supplement: Supplementary file 2 [file bmjopen-2025-111872.reviewer_comments.pdf]

PEER REVIEW HISTORY

BMJ Open publishes all reviews undertaken for accepted manuscripts. Reviewers are asked to complete a checklist review form and are provided with free text boxes to elaborate on their assessment. These free text comments are reproduced below.

ARTICLE DETAILS

Title (Provisional)

Does luteal phase support in MOH-IUI treatment improve cumulative live birth rates in couples with unexplained subfertility? Study protocol of the LUMO study: a multicenter, randomised, double blind, controlled trial with cost-effectiveness analysis

Authors

Preesman, Emma; Drechsel, Katja; Crommelin, Heleen; Broekmans, Frank; Verpoest, Willem; Broer, Simone; LUMO study group, On behalf of

---

This article was not externally reviewed at BMJ Open. Protocols that have been independently assessed prior to submission to BMJ Open are usually fast-tracked to publication on the grounds that further substantial changes will not be possible. This independent assessment will usually be external, independent review for both a substantial grant award from a non-commercial or government funder and ethics approval.
